# Utility of Machine-Learning Approaches to Identify Behavioral Markers for Substance Use Disorders: Impulsivity Dimensions as Predictors of Current Cocaine Dependence

**DOI:** 10.3389/fpsyt.2016.00034

**Published:** 2016-03-10

**Authors:** Woo-Young Ahn, Divya Ramesh, Frederick Gerard Moeller, Jasmin Vassileva

**Affiliations:** ^1^Department of Psychiatry, Virginia Commonwealth University, Richmond, VA, USA; ^2^Institute for Drug and Alcohol Studies, Virginia Commonwealth University, Richmond, VA, USA; ^3^Department of Psychology, The Ohio State University, Columbus, OH, USA; ^4^School of Nursing, University of Connecticut, Storrs, CT, USA; ^5^Department of Pharmacology and Toxicology, Virginia Commonwealth University, Richmond, VA, USA

**Keywords:** cocaine, addiction, substance dependence, impulsivity, machine learning, LASSO

## Abstract

**Background:**

Identifying objective and accurate markers of cocaine dependence (CD) can innovate its prevention and treatment. Existing evidence suggests that CD is characterized by a wide range of cognitive deficits, most notably by increased impulsivity. Impulsivity is multidimensional and it is unclear which of its various dimensions would have the highest predictive utility for CD. The machine-learning approach is highly promising for discovering predictive markers of disease. Here, we used machine learning to identify multivariate predictive patterns of impulsivity phenotypes that can accurately classify individuals with CD.

**Methods:**

Current cocaine-dependent users (*N* = 31) and healthy controls (*N* = 23) completed the self-report Barratt Impulsiveness Scale-11 and five neurocognitive tasks indexing different dimensions of impulsivity: (1) Immediate Memory Task (IMT), (2) Stop-Signal Task, (3) Delay-Discounting Task (DDT), (4) Iowa Gambling Task (IGT), and (5) Probabilistic Reversal-Learning task. We applied a machine-learning algorithm to all impulsivity measures.

**Results:**

Machine learning accurately classified individuals with CD and predictions were generalizable to new samples (area under the curve of the receiver-operating characteristic curve was 0.912 in the test set). CD membership was predicted by higher scores on motor and non-planning trait impulsivity, poor response inhibition, and discriminability on the IMT, higher delay discounting on the DDT, and poor decision making on the IGT.

**Conclusion:**

Our results suggest that multivariate behavioral impulsivity phenotypes can predict CD with high degree of accuracy, which can potentially be used to assess individuals’ vulnerability to CD in clinical settings.

## Introduction

Substance misuse is one of the biggest public health problems that have a major impact on our societies and nations. The total cost of substance use disorders (SUDs) is estimated at over $500 billion a year in the United States (1). Unfortunately, efficacious pharmacological and behavioral interventions are limited for most SUDs [but see Ref. (2)]: some medications exist for tobacco- and opiate dependence, but none are available for cocaine-, methamphetamine-, or cannabis dependence (3).

One of the key features of SUDs is *impulsivity*, defined as “a predisposition toward rapid, unplanned reactions to internal or external stimuli with diminished regard to the negative consequences of these reactions to the impulsive individual or to others.” (4, 5). Impulsivity is a multidimensional construct manifested in various ways (5, 6). Most often, impulsivity is measured by self-report measures of *trait impulsivity*, which assess impulsivity as a long-lasting personality characteristic. Impulsivity is also indexed by laboratory neurocognitive measures that most commonly assess two main processes: (1) *impulsive action* (7), i.e., compromised ability to inhibit inappropriate behaviors, and (2) *impulsive choice* (8), reflecting suboptimal choices in the face of delay contingencies or potential reward/risk. Note that these two constructs are classified differently in value-based decision-making literature (e.g., *action selection* and *valuation systems*, respectively) (9). Importantly, these various dimensions of impulsivity often do not correlate to each other (10–13), suggesting that self-report and neurocognitive tasks of impulsivity reflect different processes (14).

Previous studies show that different dimensions of impulsivity are strong predictors of drug initiation and maintenance (15–18) and are also associated with clinical treatment outcomes, such as poor treatment response and increased propensity for relapse [e.g., Ref. (19)]. Cocaine dependence (CD), among other SUDs, is strongly related to increased impulsivity (20). Cocaine-dependent individuals (CDIs) score consistently higher than healthy controls (HCs) on self-report trait impulsivity measures (19, 21, 22). Numerous neurocognitive studies also demonstrate that CDIs are characterized by both impulsive action and impulsive choice. With regards to impulsive choice, CDIs discount delayed rewards more steeply compared to HCs (23, 24); show decision-making deficits on gambling tasks, such as the Iowa Gambling task (IGT) (11, 25); and have difficulties adaptively reversing their choice preference, evidenced by increased perseverative errors on Probabilistic Reversal-Learning (PRL) tasks (26). On measures of impulsive action, CDIs show impaired response inhibition compared to HCs on neurocognitive tasks, such as the Immediate Memory Task (IMT) (21, 22) and the Stop-Signal Task (SST) (27, 28).

These results suggest that a multivariate *battery* of multiple behavioral measures could more accurately predict cocaine dependence compared to single (i.e., univariate) measures. The discovery of strong and generalizable markers of CD could help us more objectively diagnose CD and lead to important innovations in clinical settings, such as personalized prevention and intervention programs. Clearly, identifying predictors or classifiers of CD and more generally of SUDs would be a useful aim, but the field is still in its infancy (29). One of the most promising approaches is the application of machine-learning methods. Unlike conventional univariate methods, which compare healthy and psychiatric groups on each measure at a time, machine-learning (supervised learning) methods select multivariate predictive patterns of data in a way that optimizes prediction accuracy in new samples (29–31). A machine-learning approach is particularly useful when there are a large number of predictor variables (i.e., the dimensionality of feature space is large) compared to the number of samples or when our focus is on the reliability and generalizability of measures (30). However, to our knowledge, few studies have investigated SUDs from a machine-learning perspective (32, 33) and no studies have employed multiple dimensions of impulsivity as predictors in a machine-learning model.

We address these gaps by using the machine-learning method and a *battery* of behavioral measures assessing various aspects of trait impulsivity, impulsive choice, and impulsive action. The main goal of the study was to identify multivariate patterns of impulsivity that (1) best predict CD and (2) make accurate predictions that are generalizable to new samples. Here, we demonstrate that a machine-learning algorithm can identify multivariate impulsivity profiles that predict CD in new samples with high degree of accuracy.

## Materials and Methods

### Participants

The sample consisted of 23 HCs and 31 CDIs recruited from ongoing studies at the Institute for Drug and Alcohol Studies (IDAS) at Virginia Commonwealth University (VCU). Participants were recruited *via* newspaper advertisements and were initially screened by a brief telephone interview. Individuals were excluded if they indicated significant psychiatric or medical conditions, including a self-reported history of severe brain injury. Following the phone screen, eligible participants attended an in-person intake assessment session, where they were screened for psychiatric disorders using the structured clinical interview for DSM-IV (SCID-I) (34), and completed a medical history and physical examination. Information about the participants’ demographic and drug use history was also collected at the intake interview. All participants were urine tested for cocaine (benzoylecgonine), tetrahydrocannabinol (THC), opiates, amphetamine, methamphetamine, and benzodiazepines using integrated E–Z split key cup II (Innovacon Company, San Diego, CA, USA) on each visit to the clinic. Eligible cocaine-dependent participants met current DSM-IV criteria for CD; did not meet DSM-IV current dependence criteria for drugs other than cocaine, marijuana, nicotine, or alcohol; and did not have current or past medical disorders affecting the central nervous system. The cocaine-dependent sample included both treatment-seeking (*n* = 22) and non-treatment-seeking (*n* = 9) participants. The treatment seekers were part of studies, in which they received manualized cognitive behavioral therapy and were randomized to either placebo or any one or combination of the following medications: levodopa/carbidopa and/or citalopram. All data from treatment seekers were collected at intake prior to the start of medication or behavioral therapy; therefore, treatment seekers and non-treatment seekers have been grouped together for the analyses.

The HC group consisted of participants who had negative urine drug screen, negative breathalyzer test, and did not have any current or past DSM-IV axis I disorders (including substance dependence) or medical disorders affecting the central nervous system. HCs were recruited *via* similar advertising procedures as the cocaine-dependent participants.

All participants were free of alcohol at the time of testing as determined by a breathalyzer (Intoximeters, Inc., St. Louis, MO, USA). Female participants were excluded if they had a positive urine pregnancy test. All data were collected at the VCU IDAS. All participants were compensated for their participation. Participants were fully informed of the nature of the research and provided written consent for their involvement in accordance with the Declaration of Helsinki. The studies from which subject data were included were approved by the Committee for the Protection of Human Participants at VCU.

### Impulsivity Measures

#### Trait Impulsivity

##### Barratt Impulsiveness Scale-11

The Barratt Impulsiveness Scale-11 (BIS-11) is one of the most commonly used measures of trait impulsivity. The BIS-11 is a 30-item self-report scale with three oblique factors: (1) attentional/cognitive impulsivity, measuring tolerance for cognitive complexity and persistence; (2) motor impulsivity, measuring the tendency to act on the spur of the moment; and (3) non-planning impulsivity, measuring the lack of sense of the future. Total scores range from 30 to 120, with non-psychiatric controls generally scoring 50–60 (35, 36).

#### Impulsive Action

##### Immediate Memory Task

The IMT is a more complex version of the continuous performance test (CPT) designed to measure sustained attention, working memory, and response inhibition. The IMT consists of two blocks of 180 trials each. In each trial, a series of five-digit numbers (e.g., 73021) are displayed on the monitor for 0.5 s and separated by a 0.5-s interstimulus interval. Participants are instructed to respond when a five-digit number (the target stimulus) is identical to the preceding stimulus. The probability of a target stimulus is set at 33%. Three types of stimuli are presented: target (33%), catch (33%), and filler (34%). A target stimulus is a five-digit number that is identical to the preceding number. Responses to target stimuli are recorded as correct detections (or “hits”), whereas a failure to respond to a target stimulus is recorded as an omission error (or “false-negative”). A catch stimulus is a number that differs from the preceding number by only one digit (position and value determined randomly). Responses to catch stimuli are recorded as commission errors (or “false positives”). A filler stimulus is a random five-digit number that appears whenever a target or catch trial is not scheduled to appear. Responses to filler stimuli are recorded as random errors. We used four indices of impulsivity from the IMT: non-parametric discriminability (A′) that does not require the underlying distributions to be normally distributed, response bias (B_D_) (22) derived from signal detection theory (38), commission errors, and omission errors (37).

##### Stop-Signal Task

The SST measures motor impulsivity/impulsive action, defined as the inability to inhibit an already triggered motor response. In this task, participants are required to make quick key responses to visually presented “go” signals and to inhibit any response when an auditory stop signal is suddenly presented. The task consists of two phases: a practice phase of 32 trials and an experimental phase of three blocks of 64 trials per block. The primary task is a shape judgment task that requires participants to discriminate between a square and a circle. In no-signal trials (75% of the trials), only the primary-task stimulus is presented, and participants are instructed to respond to the stimulus as fast and accurately as possible. Occasionally (25% of the trials), a stop signal (750 Hz, 75 ms) is presented shortly after the stimulus onset in the primary task. In the stop-signal trials, the primary-task stimulus is followed by the auditory stop signal presented after a variable stop-signal delay (SSD), and participants are instructed to withhold their responses. The SSD is initially set at 250 ms and is adjusted continuously with the staircase tracking procedure: when inhibition is successful, SSD increases by 50 ms and when inhibition is unsuccessful, SSD decreases by 50 ms. The index of impulsivity of the SST is stop-signal reaction time (SSRT), which is the speed of the inhibitory process. SSRT was estimated by subtracting average SSD of the three blocks from median no-signal reaction time (39, 40).

#### Impulsive Choice

##### Adjusting Delay-Discounting Task

This task is designed to measure participants’ discounting rate when they are presented with the possibility of receiving a hypothetical reward determined using a choice algorithm. The task is presented on a computer screen displaying two large command buttons, one on the left and the other on the right side of the screen. The left button always displays an immediate adjusting reward (e.g., “$5.00 now”), and the right button displays a delayed reward (e.g., “$10.00 in 1 week”). Participants are exposed to a series of choices where the delay reward magnitudes are $10, $25, $100, $250, $1,000, or $2,500 at delay periods of 1 day, 1 week, 1 month, 6 months, 1 year, 5 years, or 25 years. The computer program varies the smaller, immediately available amounts across trials according to the algorithm. However, the larger delayed amount stays the same until an indifference point (i.e., where subjective values of immediate and delayed rewards are equivalent) is determined. After an indifference point is determined, the delay for the larger reward increases to the next duration. Participants are randomly assigned to complete the assessment in either ascending or descending order of delays. Non-linear regression was used to fit a hyperbolic function that has a single free parameter, *k* (discounting rate) and natural logarithm of transformation, log(*k*), was used to normalize the distribution of *k* across participants (23, 41).

##### The Kirby Monetary-Choice Delay-Discounting Questionnaire

Participants are presented with a fixed set of 27 choices between smaller, immediate rewards (SIRs) and larger, delayed rewards (LDRs). For example, in the first trial, participants are asked, “Would you prefer $54 today or $55 in 117 days?” The participant indicates which alternative s/he would prefer by circling it on the questionnaire. The order is contrived such that the trial order does not correlate to the SIR or LDR amounts, their ratio, their difference, the delay to the LDR, or the discount rate corresponding to indifference between the two rewards. An estimate of a participant’s log(*k*) was made from the participant’s pattern of choices across the 27 questions on the monetary-choice questionnaire and by fitting a hyperbolic function with logistic regression (42, 43).

##### The Iowa Gambling Task

In this computerized version of the task, participants are asked to choose between four decks of cards that result in hypothetical monetary rewards, with the goal to maximize gains. Each deck (labeled A–D) contains 60 cards. Participants must make 100 choices over the testing session. Two of the decks (A and B) are disadvantageous in that they are associated with high immediate rewards but even higher subsequent losses. Decks C and D are considered advantageous because they result in an overall long-term gain. The index of decision-making performance on the IGT is a “net score” based on the total number of cards selected from the advantageous minus the disadvantageous decks [(C + D) − (A + B)] across the 100 trials. Lower net score indicate less advantageous decision making (44).

##### The Probabilistic Reversal-Learning Task

In the Reversal-Learning task, participants are required to choose between two stimuli that differ in color. One stimulus is “correct” and the other one is “wrong,” and participants have to discover which of the two cards is correct using trial-by-trial experience and are instructed to win as much money as possible. Selection of the correct or wrong stimulus leads to positive feedback 80 or 20% of the trials, respectively. After 40 trials (stage 1), the contingencies for correct and incorrect stimuli are reversed for the subsequent 40 trials (stage 2). In other words, the correct stimulus in stage 1 becomes an incorrect stimulus in stage 2, and *vice versa* for incorrect stimulus. Only participants who pass stage 1 (eight consecutive correct responses) are included in the analysis. The ability to reverse the acquired stimulus–reward association is measured by the number of consecutive responses to the incorrect stimulus immediately following the reversal in contingencies (perseverative errors), which was uniquely impaired among chronic cocaine users in a previous study (26, 45).

### Machine-Learning Approach

To identify multivariate profiles that correctly classify and distinguish CDIs from HCs, we applied a machine-learning algorithm called the “least absolute shrinkage and selection operator” [LASSO (46)] to self-report and neurocognitive measures of impulsivity and demographic variables. Our goal was to find the precise combination of impulsivity indices that contains the minimal number of measures for the shortest testing duration, yet provides comprehensive evaluation of impulsivity and accurate group classification. The LASSO is a penalized regression method, which imposes L1 penalty indicating that the sum of absolute values of coefficients is constrained. The LASSO automatically selects variables that are important for predictions and shrinks the coefficients of unimportant variables toward 0, which improves the prediction accuracy of the regression model to new samples. We chose the LASSO in this study because it searches for the most parsimonious model compared to other penalized regression methods. Note that unlike our approach, traditional multiple regression methods are unable to select variables and fitted models are more suceptible to over-fitting.

With the exception of the LASSO and fivefold cross-validation (CV), the core procedures for generating out-of-sample prediction (penalized logistic regression) are identical to those used in a previous study (47), which provides detailed illustration of the method and step-by-step procedures. The dependent variable we aimed to classify was whether an individual meets criteria for CD or not. For computing out-of-sample predictions, we randomly split the whole dataset into a training set (67% of the data) and a test (validation) set (33% of the data). After splitting the dataset into the training (67% of the data) and test (33% of the data) sets, we fitted the LASSO model that minimized binomial deviance in the training set using 1,000 iterations of fivefold CV (i.e., divided the training set into five partitions, trained the LASSO using only four partitions, tested the model on the remaining 1 partition, and repeat the CV procedure five times, with each of the five partitions used only once as the validation data) (32, 47). Then, we made predictions on the test set based on the LASSO model estimated only with the training set. We also report prediction performance of the model on the training set for completeness.

**Figure 1:**
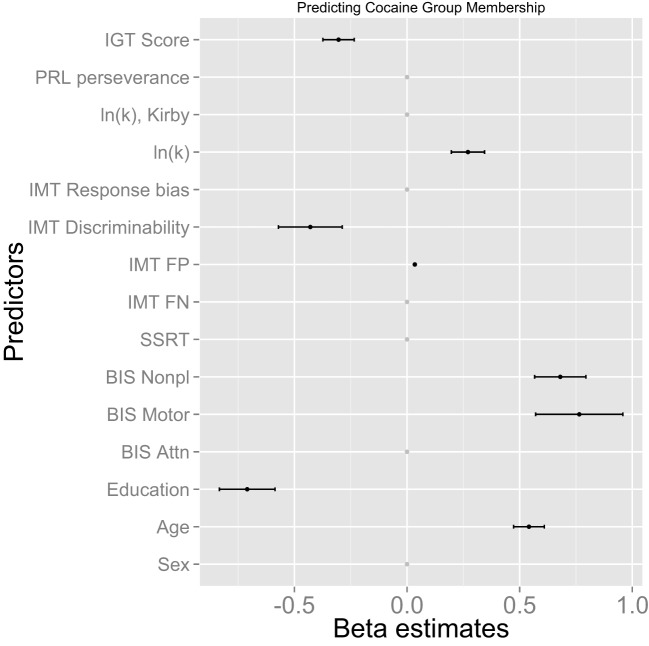
**Multivariate patterns of impulsivity measures predicting cocaine dependence**. BIS, Barratt Impulsiveness Scale; SSRT, stop-signal reaction time; IMT, Immediate Memory Task; FP, false-positive (commission) errors; FN, false-negative (omission) errors; ln(*k*), natural log of delay-discounting rate; PRL, Probabilistic Reversal Learning; IGT, Iowa Gambling Task.

For identifying predictors of cocaine dependence, we used fivefold CV across all samples, in which we used the same data as the training set and the test set. The goal was to identify predictors that are most robust across all samples. Alternatively, we could divide data into the training and test sets, identify beta coefficients of survived predictors in the training set, repeat the procedure for each randomly selected training set, and average the beta coefficients over all repetitions. Estimated beta coefficients remain essentially the same for both approaches. The mean beta coefficients over 1,000 iterations were plotted in Figure 1 where we set the mean beta coefficients of measures that survived less than 5% of 1,000 iterations to 0 for visualization purposes (their actual values are close to 0 anyway).

For the estimation of the LASSO model, we used the *glmnet* package (48). Model performance was indexed using the area under the curve (AUC) of the receiver-operating characteristic (ROC) curve, which quantifies the ability of the model to correctly assign a participant to the CD group. The pROC package (49) was used for generating a ROC curve and computing the AUC. A perfect model and a random model will score an AUC of 1.0 and 0.5, respectively. AUC values between 0.9 and 1 are considered outstanding discrimination and values between 0.8 and 0.9 are considered excellent (50). Note that in each of 1,000 iterations, we computed each individual’s “response” or fitted probabilities (0: HC and 1: CDI). A ROC curve was generated by first computing the mean fitted probabilities out of 1,000 iterations in each individual and then using the mean response and actual group information (47).

#### Robustness of Out-of-Sample Classification Accuracy

Although our procedure uses separate data for training the LASSO model and testing its out-of-sample classification accuracy, because of our relatively small sample size (*N* = 54 in total), one may wonder if the findings will generalize to new samples. To test the robustness of out-of-sample classification accuracy, we permuted the selection of training and test sets 1,000 times (while matching the proportion of CDIs and HCs in each set) and evaluated classification accuracy. In other words, we tested whether we would get similar AUC values when we differently divided data into training and test sets. In each of 1,000 repetitions, we first randomly divided data into training (67%) and test (33%) sets, and then we used the identical procedure (except that the number of iterations of fivefold CV was 100 instead of 1,000) described above to compute the distribution of AUCs on test sets. The data and codes for running the LASSO model are available at https://figshare.com/s/d4b558da3f6af69fc577.

## Results

### Subject Characteristics

Demographic and impulsivity data for all participants are presented in Table 1. The CDIs were older (*t* = −4.02; *p* < 0.001), and reported fewer years of education (*t* = 5.09; *p* < 0.001) compared to HCs. CDIs and HCs also differed in their ethnic distribution (χ^2^ = 6.28, *p* < 0.05). CDIs reported the duration of their regular cocaine use to be 16.0 ± 9.6 years. Rates of current regular tobacco use, regular cannabis use, and alcohol dependence in the cocaine-dependent groups were 74.2, 41.9, and 22.6%, respectively, which is consistent with findings from previous studies (51–53).

**Table 1:** **Characteristics of participants with complete data sets for machine-learning analyses**.

	Healthy controls (*N* = 23)		Cocaine users (*N* = 31)		Test statistic	Sig.
	Mean	SD	Mean	SD		
Age	35.39	12.1	47	7.78	−4.02	2.89E−04
Sex (% male)	43.48		64.52		1.59	n.s.
Race (% W/AA/mixed)	26.09/69.56/4.35		3.23/93.54/3.23		6.28	0.043
Education (years)	15.22	2.09	12.26	2.14	5.09	5.84E−06
Yrs of cocaine use	0.00	0.00	16.02	9.56	−9.32	2.28E−10
Yrs of cigarette use	0.00	0.00	20.29	14.03	−8.05	5.49E−09
Yrs of marijuana use	0.00	0.00	12.48	12.43	−5.59	4.39E−06
Curr. alcohol abuse (%)	0.00		12.90		1.60	n.s.
Curr. alcohol dep (%)	0.00		22.58		4.13	0.042
Curr. reg. tobacco use (%)	0.00		74.19		32.93	9.53E−09
Past reg. tobacco use (%)	0.00		12.90		9.13	0.003
Curr. reg. marijuana use (%)	0.00		41.94		15.10	1.02E−04
Past reg. marijuana use (%)	8.70		29.03		6.80	0.009
BIS attention	12.43	2.06	14.58	3.44	−2.85	0.006
BIS motor	20.48	3.29	24.35	3.96	−3.92	2.61E−04
BIS non-planning	18.87	3.82	25.9	5.78	−5.38	1.88E−06
SSRT	293.73	86.27	316.08	84.75	−0.95	n.s.
IMT omission errors	14.78	8.53	16.28	10.85	−0.57	n.s.
IMT commission errors	20.9	12.94	35.43	16.42	−3.64	0.001
IMT discriminability (A′)	0.88	0.07	0.83	0.07	2.91	0.005
IMT response bias (B_D_)	−0.15	0.39	−0.41	0.46	2.22	0.031
ln(*k*)	−6.31	1.96	−4.04	2.42	−3.81	3.66E−04
ln(*k*), Kirby	−3.92	1.35	−3.07	1.44	−2.24	0.030
PRL perseverance errors	4.74	3.26	11.55	9.70	−3.64	0.001
IGT net score	23.83	32.25	−4.87	26.98	3.46	0.001

*Independent *t*-tests were used to compute significance values, which were uncorrected for multiple comparisons. Regular use is defined as the use of a substance at least 3 days/week for the past 6 months. Curr, current; Reg, regular; W/AA, White/African-American; BIS, Barratt Impulsiveness Scale; SSRT, stop-signal reaction time; IMT, Immediate Memory Task; PRL, Probabilistic Reversal Learning; IGT, Iowa Gambling Task; n.s., non-significant (*p* > 0.10). All tests are *t*-tests except for sex, race, and % drug use variables where χ^2^ tests were used*.

### Machine-Learning Results

The training set (67% of the data) included 21 CDIs and 15 HCs, and the test set (33% of the data) included 10 CDIs and 8 HCs. Figure 1 shows multivariate patterns of impulsivity and demographic indices predicting CD, revealed by the machine-learning method. Among trait impulsivity measures, higher scores on BIS motor and BIS non-planning impulsivity predicted CD. With respect to impulsive choice, lower IGT net score and higher discounting rate predicted cocaine dependence. On impulsive action, lower IMT discriminability (A′) and higher IMT commission errors predicted CD. Among demographic variables, lower years of education and higher age predicted CD. Other measures were not critical for the prediction of CD and their coefficients shrank to 0.

**Figure 2:**
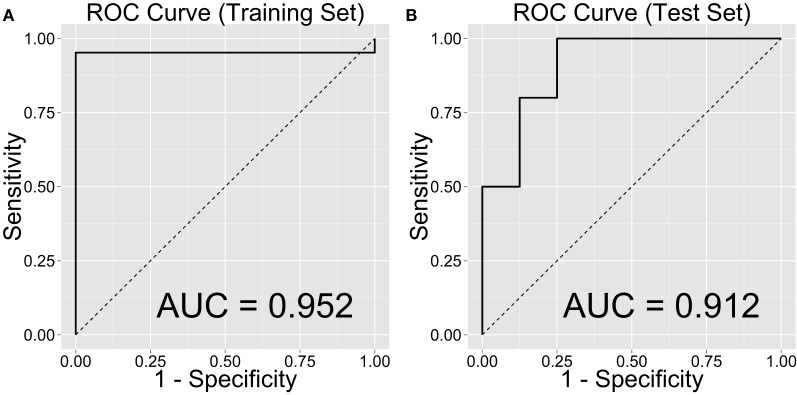
**Classification accuracy as indexed by the receiver-operating characteristic (ROC) curves and their area under the curve (AUC), separately on the (A) training and (B) test sets**.

**Figure 3:**
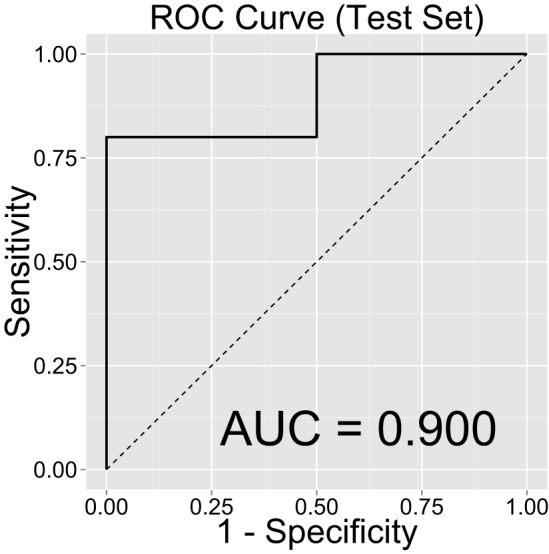
**Classification accuracy as indexed by the receiver-operating characteristic (ROC) curve and their area under the curve (AUC) on the test set, after removing the “age” regressor from the LASSO model**.

Figure 2 shows the ROC curve and its mean AUC for the prediction of CD. The AUC was 0.952 for the training set and 0.912 for the test set. Because the age difference between the two groups could be potentially arbitrary, we computed out-of-sample classification accuracy without the age variable in Figure 3 (AUC = 0.900 for the test set).

**Figure 4:**
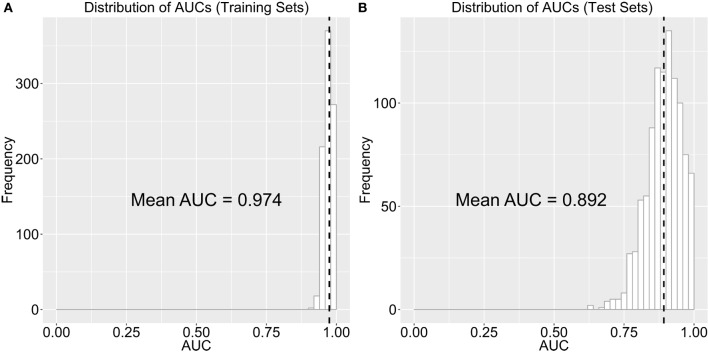
**Distribution of the area under the curve (AUC) of the receiver-operating characteristic (ROC) curve on (A) training and (B) test sets when we permutated the selection of training and test sets over 1,000 repetitions**. The black dotted line indicates the mean AUC of 1,000 repetitions.

**Figure 5:**
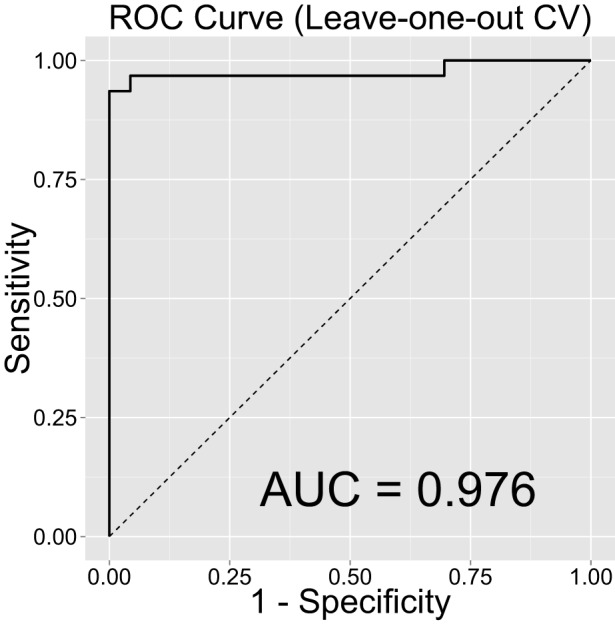
**Classification accuracy as indexed by the receiver-operating characteristic (ROC) curves and their area under the curve (AUC) using leave-one-out cross-validation**.

Note that our out-of-sample classification accuracy was reliably robust (i.e., accuracy was consistently high and above chance) even when we randomly chose training and test sets and computed AUC repeatedly over 1,000 repetitions (see Robustness of Out-of-Sample Classification Accuracy). Figure 4 shows the distribution of AUCs over 1,000 repetitions in training (Figure 4A) and test (Figure 4B) sets. Mean AUC values were 0.974 and 0.887 for the training and test sets, respectively. These results over 1,000 repetitions further increase our confidence that our findings are robust and would be generalizable to new samples. Lastly, we examined classification accuracy with leave-one-out CV [i.e., LASSO is trained on all samples except one and a prediction is made for the left-out participant and the procedure is repeated *N* (=54) times], where the AUC was even higher (0.976, Figure 5).

## Discussion

This study demonstrates that a multivariate battery of behavioral impulsivity measures can accurately predict CD using machine-learning approaches. The LASSO algorithm selected a subset of measures that were most predictive of CD (BIS motor, BIS non-planning, IMT commission errors, IMT A′, discounting rate of delayed rewards, and IGT net score), while the effects of other measures were 0 due to our use of a shrinkage method (i.e., the LASSO). While some neuroimaging studies have reported moderate to high predictive accuracy for a SUD [e.g., Ref. (33)], to our knowledge, this is one of the first machine-learning studies (32) that achieved such high out-of-sample classification accuracy (AUC = 0.912 on the test set) using only behavioral measures.

Current findings with behavioral and self-report measures of impulsivity replicate previous findings in cocaine users, suggesting that our task selection was adequate [for a complete review, see Ref. (20)]. Consistent with previous studies (19, 21, 22) in which CDIs score higher than HCs on motor, non-planning and attentional subscales of the BIS-11 when tested with univariate approaches, the non-planning and motor subscales were most predictive of CD in the current machine-learning model. On measures of impulsive action, consistent with previous studies (21, 22), CDIs had higher commission errors and poorer discriminability compared to controls but did not differ from HCs on omission errors on the IMT. We failed to observe significant group differences on the SST where previous studies have revealed mixed findings (27, 54). With regards to impulsive choice, the literature suggests that CDIs have higher *k* values on both the Monetary-Choice Delay-Discounting Questionnaire (MCDDT) and Delay-Discounting Task (DDT) compared to HCs (23, 24); ­however, only the DDT task was predictive in our machine-learning model. Consistent with the literature, CDIs also displayed lower net-scores on the IGT (11, 25). It is important to note that the LASSO tends to select only one variable if several are correlated to each other. Thus, discrepancy between *t*-test results (Table 1) and machine-learning results could be due to the nature of the LASSO algorithm, which is desirable when the goal is to reduce the number of tasks in the battery as much as possible.

**Figure 6:**
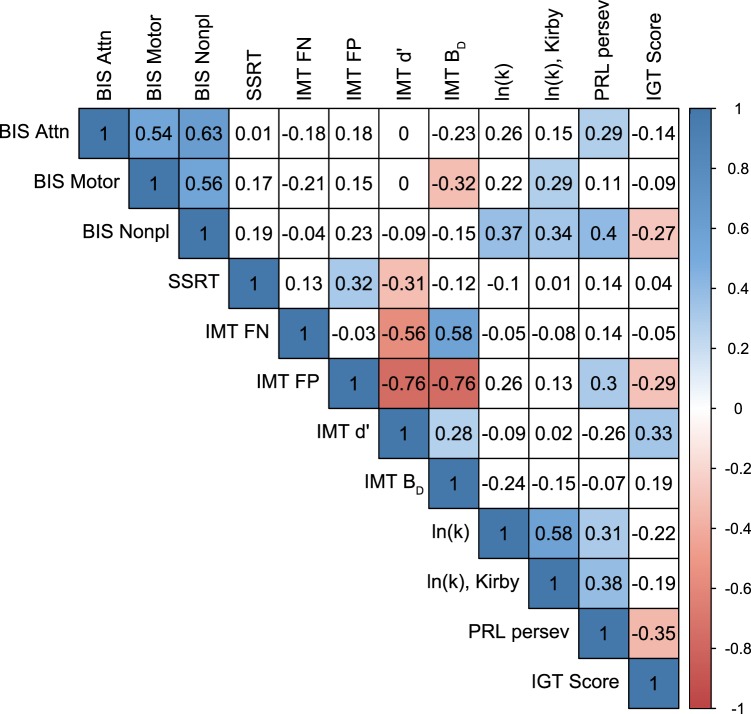
**A correlation matrix between all impulsivity measures**. Numbers in cells and color bars indicate Pearson correlation coefficients. Significant correlations (*p* < 0.05, uncorrected) are filled with blue (positive correlation) or red (negative correlation) colors. BIS, Barratt Impulsiveness Scale; SSRT, stop-signal reaction time; IMT, Immediate Memory Task; FP, false-positive (commission) errors; FN, false-negative (omission) errors; ln(*k*), natural log of delay-discounting rate; PRL, Probabilistic Reversal Learning; IGT, Iowa Gambling Task.

The current study further supports the notion that trait-like and laboratory measures of impulsivity assess non-overlapping and distinct constructs (11, 13, 55). We used both self-report trait impulsivity and laboratory impulsivity measures (impulsive choice and impulsive action), and as seen in Figure 6, they showed weak to mild correlations (except for within-measure correlations, most absolute values of Pearson correlation coefficients <0.4). The results suggest that using a battery of impulsivity measures, instead of a single measure, increases prediction accuracy.

Strong predictive markers of SUDs may innovate their prevention and treatment (56), and this study bears important implications for the development of affordable and easy-to-administer standardized assessment batteries that can evaluate individuals’ risk to CD in clinical settings. A rapidly growing literature suggests that there are several neurobiological markers of SUDs, including genetic mutations (57), dopamine D2 receptors levels (58, 59), and prefrontal-cortical function (60, 61), which serve as vulnerabilities or resilience to SUDs. These neuroimaging and genetic measures, however, are very costly, which makes their use in some clinical settings unsustainable. In contrast, our battery of impulsivity measures (BIS-11, IMT, DDT, and IGT) is cost-effective, brief, and easy to administer, requiring less than an hour to complete. In line with the “precision medicine” ­initiative (62), this approach may also have utility for personalized personality-targeted prevention and intervention programs [e.g., Ref. (56)], which can be supplemented with various cognitive remediation strategies that address the specific type of impulsivity deficits (action or choice) (63). In another work, we identify distinct substance-specific behavioral profiles for dependence on heroin vs. amphetamines by using a similar machine-learning approach (32). These findings suggest that the efficacy of prevention/intervention programs for SUDs may be improved by targeting not only individual differences in neurocognitive and personality high-risk profiles but also tailoring preventions/interventions to dissociable substance-specific high-risk profiles. In line with other studies using a multivariate machine-learning approach (33), some of the strongest predictors of CD in the current study were self-report personality measures, such as the BIS-11. We have previously demonstrated that baseline trait impulsivity (BIS-11) significantly predicts treatment retention and cocaine use among treatment-seeking cocaine users (19, 64). We believe the BIS-11 should be considered as a standard measure for studies involving SUDs (36), and we expect our multivariate battery of impulsivity including trait impulsivity measures may be helpful in predicting response to treatment.

In the current study, the predictors of the machine-learning model were all behavioral measures of impulsivity, but ample literature suggests that at least some of them could be *linked to* biological processes (29): for the IGT, making adaptive choices is related to the function of the prefrontal cortex (PFC), especially its ventromedial part (vmPFC) (65–67). Brain regions related to encoding the subjective values of delayed monetary rewards and choosing delayed rewards have been also well documented (68, 69), which include the medial/lateral PFC and ventral striatum. Future studies linking neuroimaging and surrogate measures, such as neurocognitive tasks (70) and eye movements (71), are needed for identifying such surrogate markers of CD.

The current study had several limitations. First, our sample size was relatively small and we acknowledge that larger sample sizes would result in more generalizable findings (72). However, note that out-of-sample classification accuracy was robustly high when we randomly divided data into training and test sets and repeated the procedure. Also, our sample size is greater or comparable to sample sizes in recent neuroimaging studies utilizing machine-learning methods [e.g., Ref. (73–75)]. These neuroimaging studies typically use leave-one-out CV and have substantially higher number of predictors (i.e., voxels) than our study. Still, we achieved excellent classification accuracy (AUC = 0.912 on our test set with fivefold CV and AUC = 0.976 with leave-one-out CV), which is higher or comparable to that of neuroimaging studies. Taken together, these results suggest that our findings might generalize in new samples. To our knowledge, this is one of the first studies applying a machine-learning method for the development of accurate and cost-effective behavioral markers, and the current study might promote awareness and interests in this “big data” approach in the field. The second limitation is that our current participant population abused other substances in addition to cocaine, which is representative of the patterns of drug use in the United States (76), but complicates our attempts to dissociate effects unique to CD/stimulants from those of other classes of drugs (e.g., opiates) (77). Some of our CDIs also met dependence for substances other than cocaine, including alcohol, nicotine, and marijuana; therefore, our predictors might not be specific to CD in particular. Also, our CDIs are current users, and thus, we are unsure whether their profiles are due to their current state of CD or their preexisting traits related to impulsivity and whether these profiles would persist with abstinence. Lastly, impulse control can be impacted by other conditions including subclinical depression, which we did not measure. However, we believe the impact of depression on our findings would be minimal as we excluded participants meeting DSM-IV criteria for a mood disorder. We address some of these limitations in another study (32), where we recruit individuals in Bulgaria with mono-dependence on heroin or amphetamines who are currently in protracted abstinence.

In summary, the current study demonstrates that a standardized, multivariate behavioral assessment approach to impulsivity, combined with advanced statistical learning approach could advance the field of SUDs in several important ways. With machine-learning algorithms and a multivariate approach, we can refine the design of our studies and improve the prediction accuracy for clinical outcomes. Application of this approach to longitudinal studies might enable us to identify cost-effective behavioral markers for SUDs and identify high-risk neurocognitive or personality profiles (78), which may be targeted independently by prevention/intervention programs.

## Author Contributions

W-YA analyzed the data, interpreted the results, and wrote the paper. DR performed research, interpreted the results, and wrote the paper. FM and JV interpreted the results and wrote the paper. All authors have approved the final version of the submitted manuscript.

## Conflict of Interest Statement

The authors declare that the research was conducted in the absence of any commercial or financial relationships that could be construed as a potential conflict of interest.
